# Retrograde gastric decompression and anterograde enteral nutrition feeding in retrosternal esophagectomy for esophageal cancer

**DOI:** 10.1590/1806-9282.20250171

**Published:** 2025-10-27

**Authors:** Jingrong Yang, Wenxuan Xia, Shixin Ye, Duohuang Lian, Jie Zhu, Jian Wu, Zhiyong Zeng

**Affiliations:** 1 Fujian Medical University, Fuzong Clinical Medical College – Fuzhou, China.; 2 The 900th Hospital of Joint Logistic Support Force, People's Liberation Army, Department of Cardiothoracic Surgery – Fuzhou, China.; 3 Fujian Medical University, The First School of Clinical Medicine – Fuzhou, China.

**Keywords:** Esophageal cancer, Esophagectomy, Enteral nutrition, Decompression, Postoperative care

## Abstract

**BACKGROUND:**

Postoperative care after McKeown esophagectomy remains challenging. The aim of this study was to evaluate retrograde gastric decompression and feeding as an alternative to nasogastric decompression and nasogastric-jejunal feeding.

**METHODS:**

This retrospective study analyzed 142 esophageal cancer patients undergoing McKeown esophagectomy (between June 2020 and August 2022): retrograde gastric decompression and feeding (n=74) vs. nasogastric-jejunal (n=68). Outcomes included operative parameters, complications, and recovery metrics.

**RESULTS:**

Retrograde gastric decompression and feeding required longer operative time (183.0±41.7 vs. 169.4±32.6 min, p=0.031) but showed comparable blood loss, R0 resection rates (95.9 vs. 97.1%), and lymph node yield. Gastric tube retention was shorter with retrograde gastric decompression and feeding (3.2±1.6 vs. 3.6±1.4 days). Complication rates (anastomotic leak: 10.8 vs. 10.3%; respiratory: 16.2 vs. 16.2%) and in-hospital mortality (1.4 vs. 1.5%) were similar. Tube-related complications trended lower with retrograde gastric decompression and feeding (5.4 vs. 10.3%, p=0.276).

**CONCLUSION:**

Retrograde gastric decompression and feeding is a safe, effective method for enteral nutrition and decompression post-esophagectomy.

## INTRODUCTION

Esophageal cancer remains a major global malignancy, with approximately 511,000 new cases and 445,000 deaths annually^1^. For resectable cases, McKeown esophagectomy via retrosternal approach has emerged as a preferred surgical alternative to conventional open procedures, offering reduced operative trauma and complication rates^2,3^.

Despite these benefits, esophageal cancer management requires meticulous postoperative care to mitigate complications associated with conventional tube placements^4^. Current practice predominantly utilizes nasogastric-jejunal (NGJ) tubes^5^, which carry substantial risks of nasal injury, tube dislodgement, and sinusitis^6^.

We developed a modified retrograde gastric decompression and feeding (RGDF) technique during McKeown procedures. By establishing a gastrostomy fistula for simultaneous decompression and jejunal feeding, this approach aims to eliminate nasal trauma while maintaining nutritional efficacy.

This study retrospectively evaluated the feasibility, safety, and short-term efficacy of the RGDF technique in patients undergoing McKeown-type esophagectomy. Specifically, we aimed to assess its impact on postoperative recovery, complication rates, and overall patient outcomes.

## METHODS

### Patients

This retrospective study analyzed 142 esophageal cancer patients undergoing McKeown esophagectomy with retrosternal reconstruction (between June 2020 and August 2022). Patients were stratified into RGDF (n=74) and NGJ (n=68) groups based on postoperative management. Inclusion criteria required: histopathologically confirmed esophageal cancer, clinical stage T1-3M0 (AJCC 7th ed.^7^), adequate cardiopulmonary function, and absence of severe comorbidities or adhesions. The Ethics Committee of Fuzhou General Hospital approved the protocol, with written consent obtained.

### Surgical procedures

**Figure 1:**
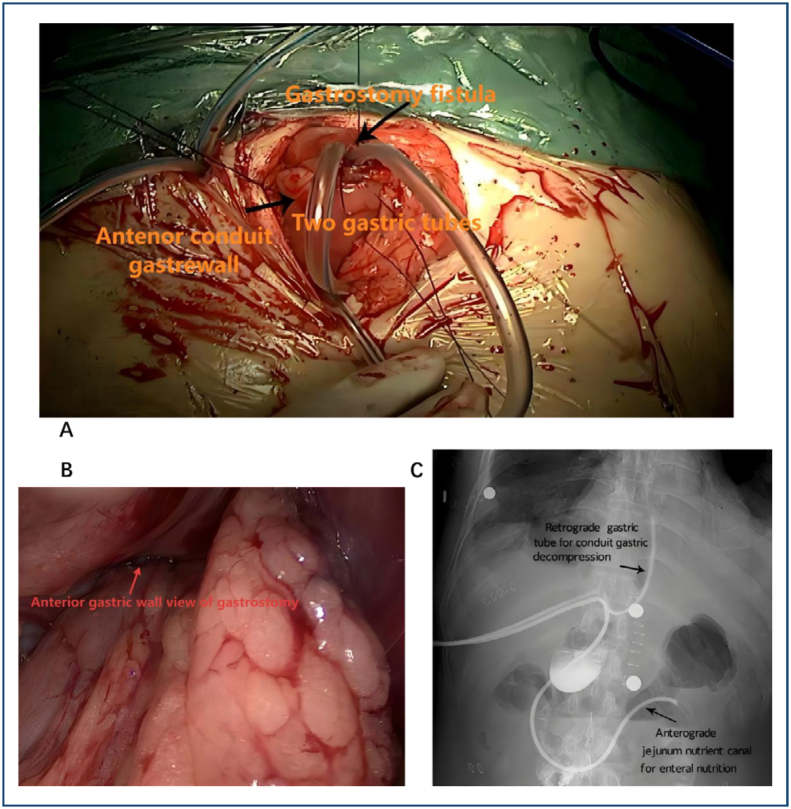
Gross anterior view (A), intraperitoneal view (B), and plain abdominal radiograph (C) after tube placement.

The procedure followed Yang et al.'s VATS approach^8^, comprising three phases:

(1) Thoracic: pulmonary ligament division, esophageal mobilization, azygos vein ligation, and mediastinal lymphadenectomy; (2) Abdominal: laparoscopic gastric mobilization with lymph node dissection and creation of a 40–50 mm gastric conduit; and (3) Cervical: left neck incision with esophageal transection and end-to-end anastomosis (EEA). Figure 1 shows the gross anterior view (1A), intraperitoneal view (1B), and plain abdominal radiograph (1C) after tube placement.

The RGDF group received an additional gastrostomy: Two tubes (16 Fr, 125 cm in length, 5.3 mm in diameter; TERUMO Medical Products Co., Ltd., Hangzhou, China) were inserted through a 1 cm incision, 3 cm to the right of the midline. A double purse-string suture was placed 5 cm proximal to the pylorus, with one tube advanced 10 cm retrogradely and another 40 cm anterogradely into the jejunum. The placement was confirmed radiographically.

The NGJ group received standard nasogastric (35–40 cm) and nasojejunal (80–100 cm) tube placement.

Intraoperative and postoperative parameters were collected to evaluate surgical outcomes, including blood loss, operative time, extent of resection (R0, R1, and R2), harvested lymph nodes, and recovery metrics such as intensive care unit (ICU) stay, hospital stay, and duration of gastric and feeding tube placement. Postoperative complications were categorized into surgical and tube-related complications. In-hospital mortality was also recorded. Postoperative drainage volumes were recorded, and pain scores were evaluated by visual analog scale methods.

### Postoperative management

Both groups received standardized decompression using Fr14 negative-pressure suction devices (Shandong Baiduoan Medical). Enteral nutrition was initiated on postoperative day (POD) 1 through a stepwise protocol: Phase 1 (POD1): 5% glucose solution at 20 mL/h; Phase 2 (POD2–3): Transition to Peptisorb^®^ liquid (25 mL/h); Phase 3 (POD4–7): Progressive escalation to 200 mL/h.

Nutritional tubes were flushed with 30 mL warm water before/after each infusion to maintain patency. Patients achieving stable tolerance of 200 mL/h feeding without complications (e.g., diarrhea requiring rate adjustment) were discharged. All enteral tubes were electively removed 14 days post-discharge following outpatient confirmation of oral intake adequacy.

### Statistical analysis

Statistical analyses used SPSS 25 with continuous variables presented as mean±standard deviation (SD) (t-test/Mann-Whitney U test) and categorical variables as percentages (χ^2^/Fisher's exact test). Significance threshold was p<0.05. Post-hoc power analysis assessed sample adequacy.

## RESULTS

### Baseline demographics and clinicopathological features

The study included 142 patients (RGDF=74 and NGJ=68) with comparable baseline profiles (Table 1). Both groups predominantly comprised males (RGDF 70.3% vs. NGJ 64.7%, p=0.501) and squamous carcinoma histology (93.2 vs. 94.1%). Tumor distribution showed similar patterns, with the middle esophagus being the most frequent (58.1 vs. 61.8%). Tumor-Node-Metastasis (TNM) staging revealed 95.9% of RGDF and 90% of NGJ patients in stages I–III (p=0.129).

**Table 1:** Demographic and clinicopathological characteristics of patients.

Parameters		RGDF group n=74	NGJ group n=68	p-value
Demography				
	Onset age (mean±SD)	(58.1±13.1) years	(53.7±11.2) years	0.100
	Male, n (%)	52 (70.3%)	44 (64.7%)	0.501
	Female, n (%)	22 (29.7%)	24 (35.3%)	
Location of lesion				
	Upper third, n (%)	18 (24.3%)	17 (25.0%)	0.773
	Middle third, n (%)	43 (58.1%)	42 (61.8%)	
	Lower third, n (%)	13 (17.6%)	9 (13.2%)	
Histological type				
	Squamous carcinoma, n (%)	69 (93.2%)	64 (94.1%)	0.935
	Adenocarcinoma, n (%)	3 (4.1%)	2 (2.9%)	
	Others, n (%)	2 (2.7%)	2 (2.9%)	
Depth of tumor invasion				
	Tis, n (%)	3 (4.1%)	1 (1.5%)	0.912
	T1, n (%)	11 (14.9%)	9 (13.2%)	
	T2, n (%)	31 (41.9%)	30 (44.1%)	
	T3, n (%)	27 (36.5%)	26 (38.2%)	
	T4, n (%)	2 (2.7%)	2 (2.9%)	
Lymphatic metastasis				
	N0, n (%)	33 (44.6%)	30 (45.6%)	0.797
	N1, n (%)	30 (40.5%)	28 (41.2%)	
	N2, n (%)	8 (10.8%)	9 (13.2%)	
	N3, n (%)	3 (4.1%)	1 (1.5%)	
TNM stage				
	Tis, n (%)	3 (4.1%)	1 (1.5%)	0.129
	I, n (%)	25 (33.8%)	29 (42.6%)	
	II, n (%)	30 (40.5%)	32 (47.1%)	
	III, n (%)	16 (21.6%)	6 (8.8%)	

RGDF: retrograde gastric decompression and feeding; NGJ: nasogastric-jejunal; SD: standard deviation; TNM: tumor, node, metastasis.

### Peri-operative outcomes and complications

RGDF required longer operative time (183.0±41.7 vs. 169.4±32.6 min, p=0.031) but showed comparable blood loss (196.9±51.6 vs. 185.5±41.9 mL) and R0 rates (95.9 vs. 97.1%). Both groups had similar lymph node yield (13.7±5.1 vs. 12.5±3.9), hospital stay (9.8±2.7 vs. 10.2±3.1 days), and gastric decompression duration (3.2±1.6 vs. 3.6±1.4 days, p=0.116). There was no difference in the mean drainage time between the RGDF and NGJ groups (2.03±0.88 vs. 1.98±1.23). The pain score of the NGJ group was significantly higher than that of the RGDF group (2.96±1.78 vs. 3.67±1.23) but with only a small increase. Postoperative complications within 3 weeks are summarized in Table 2. The overall incidence of complications was similar between the RGDF and NGJ groups, with anastomotic leakage (10.8 vs. 10.3%), respiratory complications (16.2 vs. 16.2%), and respiratory infections (10.8 vs. 10.3%) being the most frequent. RGDF showed a clinically relevant 48% reduction in tube-related complications (5.4 vs. 10.3%, p=0.276). In the RGDF group, the most common tube-related complications were obstruction (1.4%), wound infection (1.4%), and leakage around the nutrient canal (2.7%). In the NGJ group, tube dislodgement (4.4%), nasal alar necrosis (1.5%), and nasosinusitis (2.9%) were more frequently observed. Gastric emptying disorders were not observed in either group. Mortality rates were equivalent (1.4 vs. 1.5%) (Table 2).

**Table 2:** Peri-operative outcomes and complications.

Parameters		RGDF group, n=74	NGJ group, n=68	p-value
Blood loss (mL), mean±SD		196.9±51.6	185.5±41.9	0.153
Operation time (min), mean±SD		183.0±41.7	169.4±32.6	0.031*
Surgical resection status				
	R0 resection, n (%)	71 (95.9%)	66 (97.1%)	0.877
	R1 resection, n (%)	1 (1.4%)	1 (1.5%)	
	R2 resection, n (%)	2 (2.7%)	1 (1.5%)	
Harvested lymph node, mean±SD		13.7±5.1	12.5±3.9	0.120
Days of keeping a gastric tube for decompression, mean±SD		3.2±1.6	3.6±1.4	0.116
Days of keeping a feeding tube, mean±SD		23.7±7.6	22.5±8.1	0.364
ICU stay (days), mean±SD		3.2±1.0	3.5±1.2	0.107
LOHS (days), mean±SD		9.8±2.7	10.2±3.1	0.413
Postoperative drainage time (days), mean±SD		2.03±0.88	1.98±1.23	0.7797
Postoperative pain score		2.96±1.78	3.67±1.23	0.007*
Surgical complications, n (%)				
	Recurrent laryngeal nerve injury	4 (5.4%)	3 (4.4%)	1.000
	Anastomotic leak	8 (10.8%)	7 (10.3%)	0.920
	Anastomotic stenosis	6 (8.1%)	4 (5.9%)	0.747
	Postoperative hemorrhage	1 (1.4%)	1 (1.5%)	1.000
	Chylothorax	2 (2.7%)	1 (1.5%)	1.000
	Cervical wound infection	1 (1.4%)	1 (1.5%)	1.000
	Respiratory complications	12 (16.2%)	11 (16.2%)	1.000
	Respiratory infections	8 (10.8%)	7 (10.3%)	0.920
	Respiratory failure	2 (2.7%)	1 (1.5%)	1.000
	Gastric emptying disorders	0 (0.0%)	0 (0.0%)	1.000
	Arrhythmia and other cardiac complications	3 (4.1%)	3 (4.5%)	1.000
Tube-related complications, n (%)		4 (5.4%)	7 (10.3%)	0.276
	Tube obstruction	1 (1.4%)	1 (1.5%)	1.000
	Wound infection	1 (1.4%)	Null	Null
	Peripheral leakage of the nutrient canal	2 (2.7%)	Null	Null
	Tube falling off	0 (0%)	3 (4.4%)	0.107
	Necrosis of the nasal alar	Null	1 (1.5%)	Null
	Nasosinusitis	Null	2 (2.9%)	Null
	In-hospital mortality, n (%)	1 (1.4%)	1 (1.5%)	1.000

RGDF: retrograde gastric decompression and feeding; NGJ: nasogastric-jejunal; SD: standard deviation; ICU: intensive care unit; LOHS: length of post-hospital stay.

* p<0.05.

## DISCUSSION

Surgical resection remains the cornerstone treatment for resectable esophageal cancer, yet postoperative management of gastric conduit decompression and enteral nutrition continues to pose significant challenges. This study addresses the limitations of conventional NGJ tube placement by proposing RGDF as a dual-function alternative. Our findings demonstrate that RGDF, when applied to McKeown esophagectomy via the retrosternal route, achieves surgical outcomes comparable to those of NGJ while potentially reducing tube-related complications.

Nasogastric drainage is commonly used after esophagectomy to prevent gastric distension, which can significantly increase postoperative complications such as aspiration pneumonitis and mechanical strain on the anastomotic stoma. These issues may result in conduit gastric ischemia and impaired healing of the anastomosis^9^. Despite their benefits, nasogastric tubes are associated with various complications, including pharyngitis, sinusitis, nasal alar necrosis, accidental dislodgment, gastrointestinal bleeding, and aspiration pneumonia^10^. During the perioperative period, maintaining adequate nutrition is equally important, as the majority of patients with esophageal cancer present with preoperative malnutrition. Enteral nutrition has been shown to significantly reduce postoperative complications, including infectious complications such as anastomotic fistulas and intra-abdominal abscesses^11-13^. Common postoperative enteral nutrition methods include oral intake, nasointestinal tubes, and jejunostomy. However, each method has its limitations: early oral intake may increase the risk of anastomotic fistulas and aspiration pneumonia^14^; nasointestinal tubes can impede effective coughing and compromise pulmonary hygiene^15^; and jejunostomy may lead to complications such as wound infection, volvulus, hernia, and bowel obstruction^16^.

The rationale for selecting the McKeown esophagectomy with retrosternal reconstruction lies in its anatomical suitability for RGDF, particularly in the context of esophageal cancer epidemiology in China. Middle esophageal cancer accounts for the majority of cases in China^17^, and the retrosternal pathway in McKeown esophagectomy provides optimal exposure for mid-thoracic tumors while minimizing anastomotic tension. This anatomical alignment not only facilitates radical resection of middle-third lesions but also enables simultaneous gastrostomy-based decompression and feeding tube placement. Specifically, the retrosternal route offers direct access to the gastric conduit, allowing for precise positioning of retrograde and anterograde tubes without compromising thoracic cavity integrity—a critical advantage for RGDF implementation. This approach eliminates nasal trauma—a well-documented drawback of NGJ tubes^10^—and aligns with prior studies advocating retrograde decompression as a safer alternative^18^. Notably, our cohort exhibited a clinically relevant 48% reduction in tube-related complications with RGDF (5.4 vs. 10.3%, p=0.276), albeit without statistical significance. This trend mirrors findings by Puri et al.^18^, who reported fewer complications with retrograde jejunogastric decompression compared to nasogastric drainage. However, our study extends these observations by integrating enteral feeding into the same gastrostomy site, a novel modification that may streamline postoperative care.

Furthermore, the shorter duration of gastric tube retention in the RGDF group (3.2±1.6 vs. 3.6±1.4 days) may contribute to earlier patient mobilization and reduced anastomotic strain, though direct causal evidence remains to be established. Mechanistically, retrograde decompression combined with anterograde feeding may synergistically promote bowel function recovery through gravitational drainage and peristaltic stimulation—a hypothesis supported by the absence of gastric emptying disorders in both groups.

Despite these benefits, RGDF is not without limitations. The procedure required a longer operative time (183.0±41.7 vs. 169.4±32.6 min, p=0.031), likely due to the technical complexity of dual-tube placement. Challenges such as pyloric sphincter spasm and anatomical angulation between the conduit stomach and duodenum occasionally hindered tube advancement, underscoring the need for advanced surgical expertise. Additionally, while our data suggest RGDF's safety, the retrospective design introduces potential selection bias, and the lack of long-term nutritional follow-up limits conclusions about sustained efficacy. Future prospective studies with standardized nutritional assessments are warranted to validate these findings.

RGDF appears to be a safe and feasible method for enteral nutrition and decompression following McKeown esophagectomy. Its ability to mitigate nasal complications while maintaining nutritional efficacy positions it as a promising alternative to NGJ, particularly in centers proficient in minimally invasive techniques. Further research should focus on cost–benefit analyses, patient-reported outcomes, and comparisons with jejunostomy-based approaches to refine postoperative care protocols.

## CONCLUSION

RGDF is a safe, simple, and effective method for the enteral nutrition and decompression of the conduit stomach in patients who have undergone esophagectomy.

## Data Availability

The datasets generated and/or analyzed during the current study are available from the corresponding author upon reasonable request.
